# Low-cost treated lignocellulosic biomass waste supported with FeCl_3_/Zn(NO_3_)_2_ for water decolorization

**DOI:** 10.1038/s41598-022-20883-4

**Published:** 2022-09-30

**Authors:** Asiyeh Kheradmand, Mehrdad Negarestani, Afsaneh Mollahosseini, Hadi Shayesteh, Hamidreza Farimaniraad

**Affiliations:** 1 Department of Civil and Environmental Engineering, Iran University of Science and Technology (IUST), Narmak, Tehran, Iran; 2 Research Laboratory of Spectroscopy and Micro and Nano Extraction, Department of Chemistry, Iran University of Science and Technology (IUST), Narmak, Tehran, Iran; 3 School of Chemical, Petroleum and Gas Engineering, Iran University of Science and Technology (IUST), Narmak, Tehran, Iran; 4 Department of Environmental Engineering, Graduate Faculty of Environment, University of Tehran, Tehran, Iran

**Keywords:** Biogeochemistry, Environmental sciences, Chemistry, Engineering, Materials science

## Abstract

Dye pollution has always been a serious concern globally, threatening the lives of humans and the ecosystem. In the current study, treated lignocellulosic biomass waste supported with FeCl_3_/Zn(NO_3_)_2_ was utilized as an effective composite for removing Reactive Orange 16 (RO16). SEM/EDAX, FTIR, and XRD analyses exhibited that the prepared material was successfully synthesized. The removal efficiency of 99.1% was found at an equilibrium time of 110 min and dye concentration of 5 mg L^−1^ Adsorbent mass of 30 mg resulted in the maximum dye elimination, and the efficiency of the process decreased by increasing the temperature from 25 to 40 °C. The effect of pH revealed that optimum pH was occurred at acidic media, having the maximum dye removal of greater than 90%. The kinetic and isotherm models revealed that RO16 elimination followed pseudo-second-order (R^2^ = 0.9982) and Freundlich (R^2^ = 0.9758) assumptions. Surprisingly, the performance of modified sawdust was 15.5 times better than the raw sawdust for the dye removal. In conclusion, lignocellulosic sawdust-Fe/Zn composite is promising for dye removal.

## Introduction

**Figure 1:**
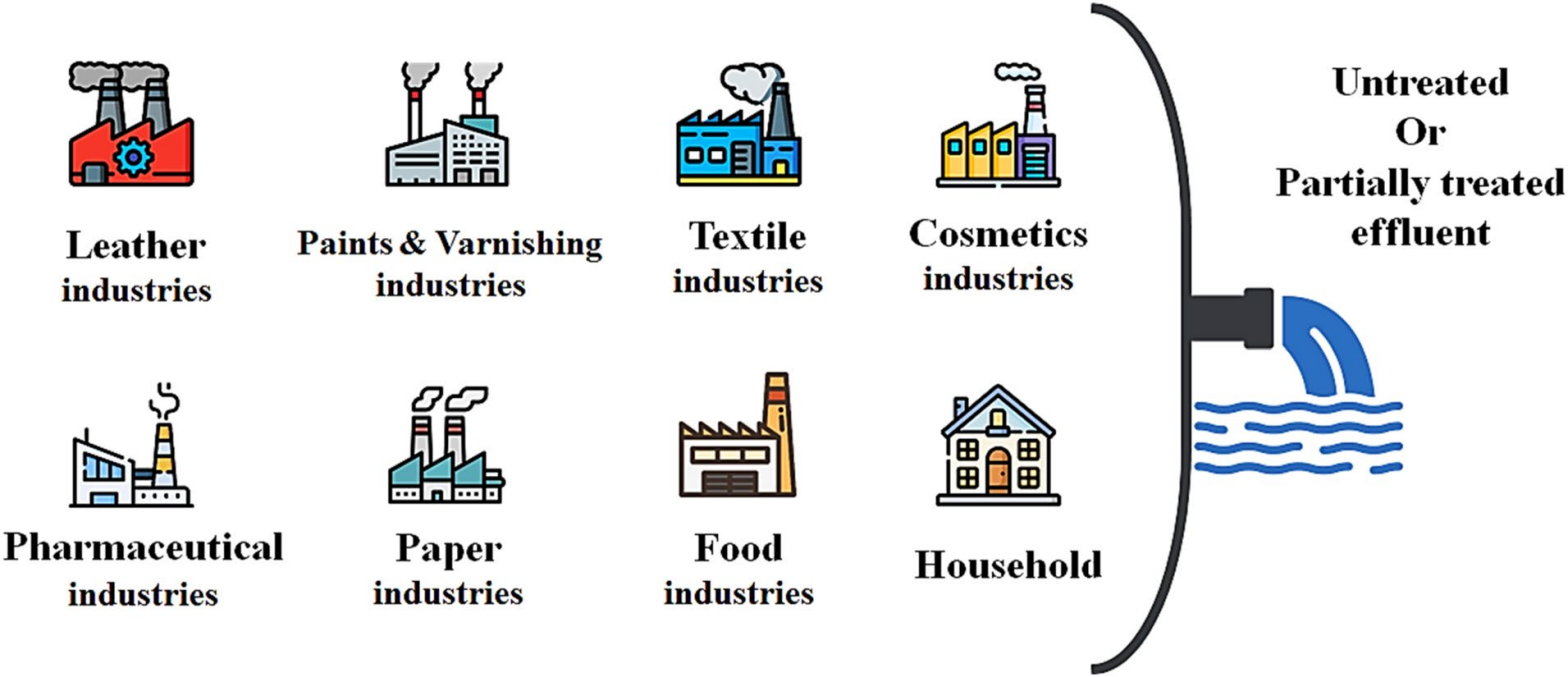
Different sources of discharging dyes into the aquatic environment.

Industries release dye effluent into the environment. Figure 1 illustrates the sources of dye pollutants in water bodies^1^. The direct discharge of dye-containing effluent into natural water bodies adversely affects the photosynthetic activity of aquatic ecosystems. Due to its appearance in the food chain, and increasing biochemical and chemical oxygen demand, dye pollution inhibits plants growth.Moreover, the excess amount of dye is carcinogenic threatening human-being lives. Therefore, removing dye from industries wastewater before discharge to waterways is high on the environmental agenda^2–4^. Several separation methods have been used to treat dye effluent including membrane separation^5^, adsorption^6^, chemical oxidation^7^, and coagulation–flocculation^8^. The mentioned methods have merits and drawbacks while among all, adsorption seems to be a promising method for the treatment of Industries wastewater. Although many scholars have already proposed countless adsorbents and composites, it is attempted to use natural-based materials, mainly to their non-toxicity and inexpensiveness.

Farmlands and jungles are invaluable natural, environmentally friendly, resistant, and renewable timber sources^9,10^. Sawdust is a lignin-based waste product of timbers majorly constituted of cellulose, lignin, acids, sugar, and other organic materials^11^. In recent years, wood waste has created major environmental issues including methane emissions and carbon monoxide emissions. Therefore, it must be removed from waterways and the atmosphere before it enters. High carbon content in wood waste results in methane production which is one of the major trade-offs that needs to be dealt with before enhancing global warming^12,13^. Wood waste buried in landfills produces even more pollutants as well as sawdust burning, which emits both carbon monoxide and nitrogen dioxide, so finding an alternative solution is of utmost importance. As a solution, it can be proposed that sawdust waste can be utilized as an efficient, natural, and low-cost adsorbent for water purification. In other words, lignocellulosic sawdust utilization could simultaneously bring two advantages: (1) waste management and (2) water decontamination. Note that raw natural-based materials (such as sawdust) may possess low adsorption capacity for some organic compounds^14^. Many studies have investigated utilizing sawdust waste as a bio-sorbent for the removal of contaminants from the environment is a feasible answer to environmental issues^15^.

Singh et al.^16^ found a low adsorption capacity of 0.2636 mg g^−1^ for dye removal by sawdust. Alidadi et al.^17^ stated that chemical modification could effectively increase the performance of sawdust. Zhou et al.^18^ could effectively increase the sawdust applicability via modification. It is therefore recommended to modify the surface of the lignocellulosic sawdust to increase its performance.

Among many chemical reagents, ferric chloride is known as relatively safe and cheap reagent that has been extensively used in many water and wastewater treatment plants as the coagulant/flocculent. Large surface area, microporous structure, high adsorption affinity and capacity, cost-effectiveness, and high anion exchange capability are the main advantages of the Fe-based adsorbents^19–21^. More importantly, Fe is able to reduce the amount of negative charge on the surface^22^, providing better conditions for the removal of anionic species. Similarly, Zn-modified composites have also been popular in recent years for the removal of pollutants^23^. de Bruijn et al.^24^, Chaichanawong et al.^25^ and Nakarmi et al.^26^ proved that Zn could modify their composite, resulting in better usability. Coupling Fe and Zn simultaneously could provide excellent conditions for the removal of pollutants from water. To date, no report has been identified using treated lignocellulosic sawdust waste supported with FeCl_3_/Zn(NO_3_)_2_ for the removal of RO16 dye.

Hence, in the current study for the first time lignocellulosic Sawdust-Fe/Zn composite was prepared for the removal of RO16 dye from aquatic media. Accordingly, the removal process was optimized and was investigated by different kinetics and isotherms.

## Experimental procedure

### Chemicals and equipment

**Figure 2:**
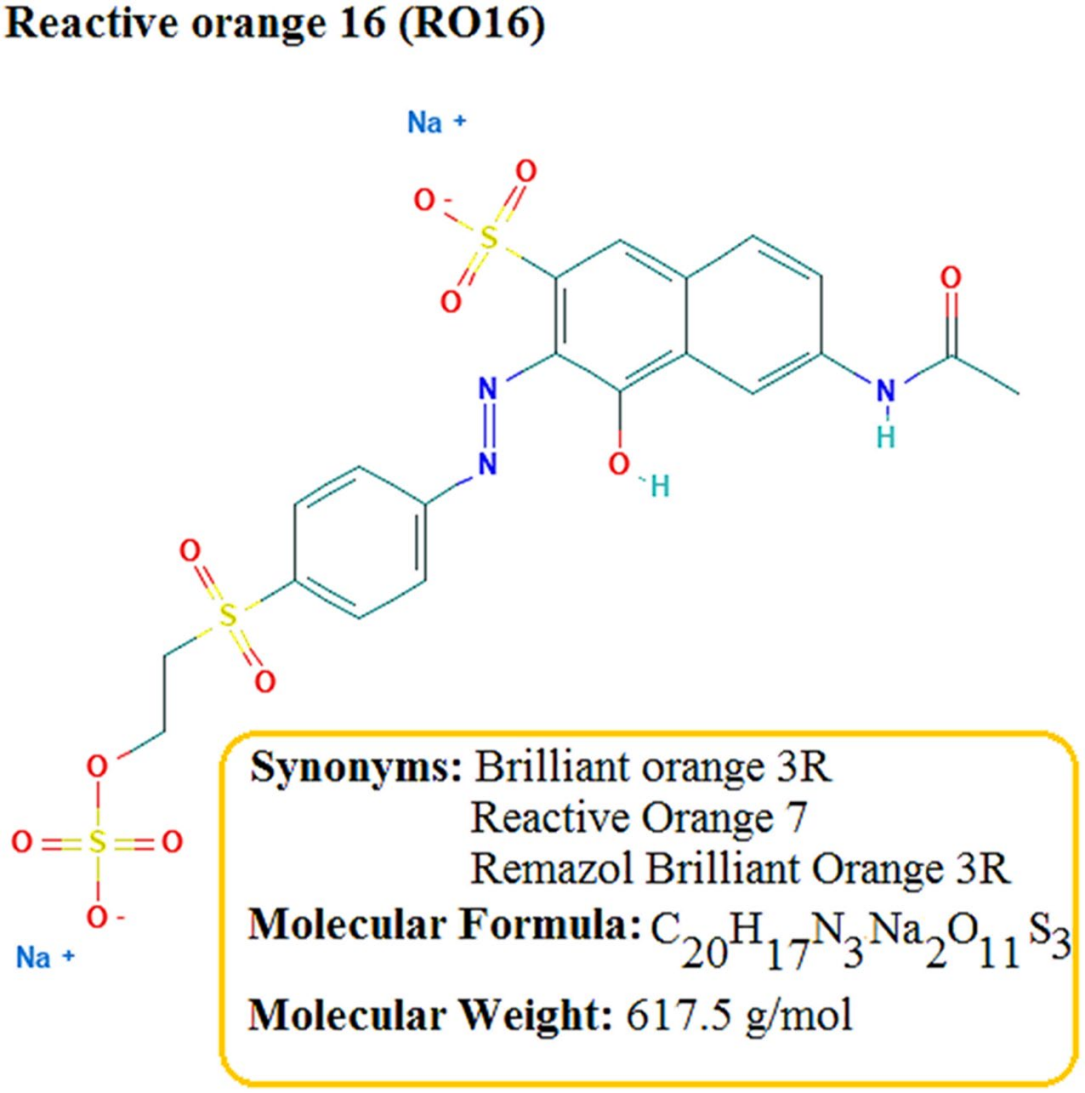
Structure and properties of RO16 dye.

Reactive orange 16 (99%) was bought from Avrin Chemistry Company, Iran (Fig. 2). All other chemicals, including iron(III) chloride (97%), zinc nitrate (98%), sodium hydroxide (97%), and sulfuric acid (98%) were all provided from Merck, Germany, having high purity. The water utilized in all the experiments was deionized (DI) water. The stock and working standard solutions were stored at 4 °C. All reagents were used without further purification.

Scanning electron microscopy (SEM) model TESCAN-Vegu II was utilized to observe the surface structure of lignocellulosic sawdust and the modified adsorbent. Also, Fourier transform infrared spectroscopy (FTIR) model Tensor 27 Bruker and X-ray diffraction (XRD) model BOUREVESTNIK DRON-8 X-ray were employed.

### Adsorbent preparation

Sawdust waste was taken from a carpentry shop in Tehran, Iran. The treatment procedure was almost followed by the previous articles^27,28^. Firstly, it was washed several times with hot water to remove surface adhered particles or any other impurities. Then it was dried at 70 °C for 48 h. After that, sawdust was treated with 1 N H_2_SO_4_ and 1% NaOH for 24 h. The prepared after was then dried again and called treated lignocellulosic sawdust. In the last step, it was sieved (No.12 ≈ 1.7 mm) to have particles with semi-sizes.

**Figure 3:**
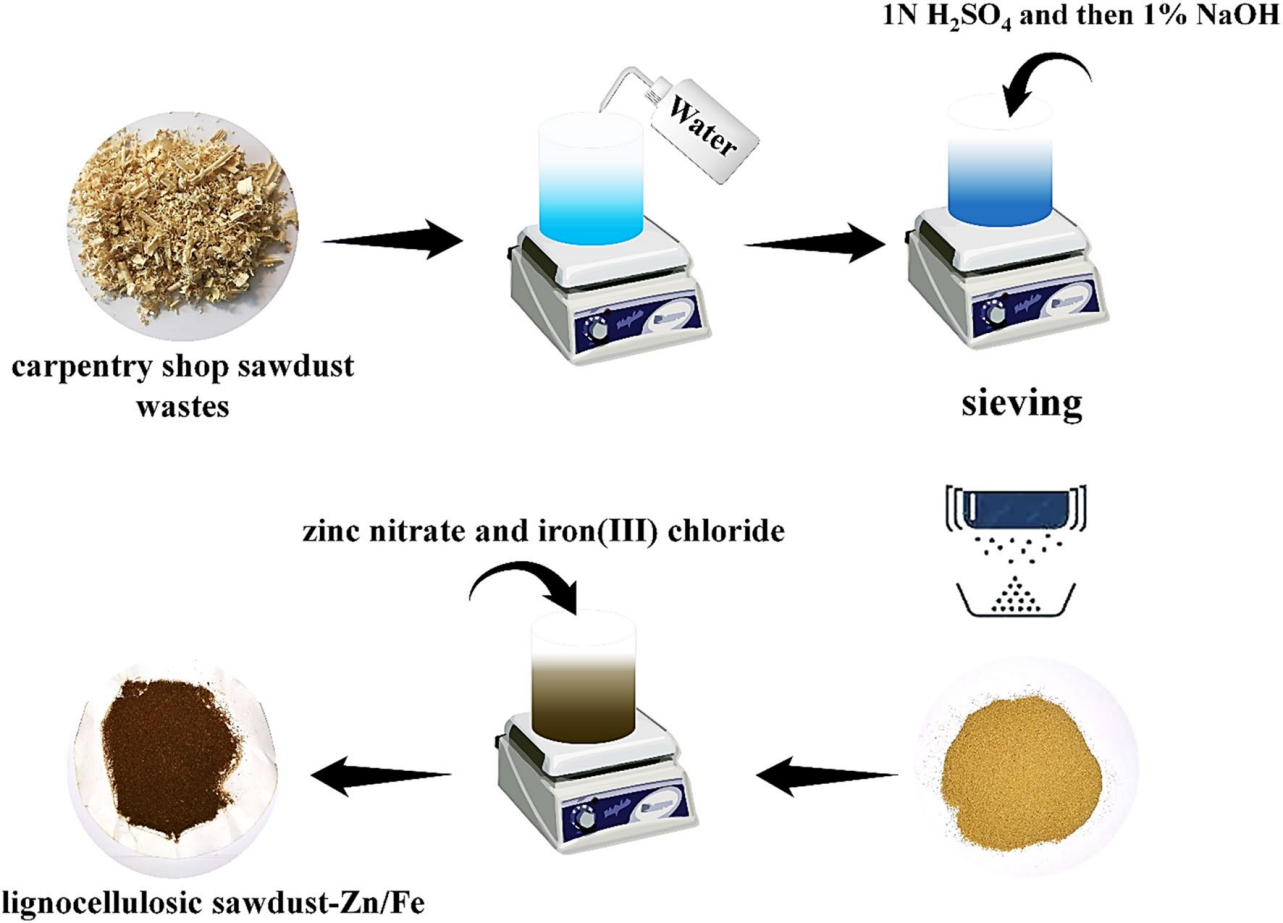
The synthesis schematic of lignocellulosic sawdust-Fe/Zn.

In order to modify the treated lignocellulosic sawdust with Fe/Zn, the coprecipitation route was used as described in the literature^29,30^. Firstly, treated lignocellulosic sawdust (1 g) was added to 60 mL water, and then the solution was agitated for 6 h. Thereafter, zinc nitrate (24 g) and iron(III) chloride (8 g) were added to the previous solution and agitated for 24 h. The initial pH of the solution was kept at 8. The resulting precipitate was filtered and washed using distilled water several times and dried at 60 °C. The final material was a modified adsorbent called lignocellulosic sawdust-Fe/Zn composite. The synthesis schematic of lignocellulosic sawdust-Fe/Zn illustrates in Fig. 3.

### Adsorption procedure

In the current investigation, removal of RO16 dye by lignocellulosic sawdust-Fe/Zn composite was tested in order to find the maximum dye removal efficiency. All experiments were conducted at a 100 mL Erlenmeyer flask. To maximize the adsorption efficiency, the influence of contact time (0–160 min), initial dye concentration (5, 10, 20, 40, 50 mg L^−1^), adsorbent dosage (5, 10, 15, 20, 30 mg), initial pH (2, 4, 6, 8, 10), agitation speed (0, 50, 100, 200, 300 rpm), and temperature (25, 30, 35, 40 °C) were investigated. Once the adsorption process was completed, 3 mL of the solution was inserted into UV-spectrophotometer to determine the remained RO16 concentration at the λ_max_ 494 nm. The amount of RO16 adsorbed at equilibrium q_e_ (mg g^−1^), and the removal percentage were calculated by Eqs. (1) and (2), respectively:

1 $${q}_{e}=\frac{({C}_{0}-{C}_{e})\times V}{m}$$

2 $$\mathrm{Removal efficiency }(\mathrm{\%})= \frac{{C}_{0}-{C}_{e}}{{C}_{0}}\times 100$$

where *C*_*0*_ (mg L^−1^) and *C*_*e*_ (mg L^−1^) are the initial and the equilibrium concentration of dye, respectively, and V is the volume of the dye solution (L), and m is the composite weight (g) added to the dye solution.

## Result and discussion

### Adsorbent characterization

**Figure 4:**
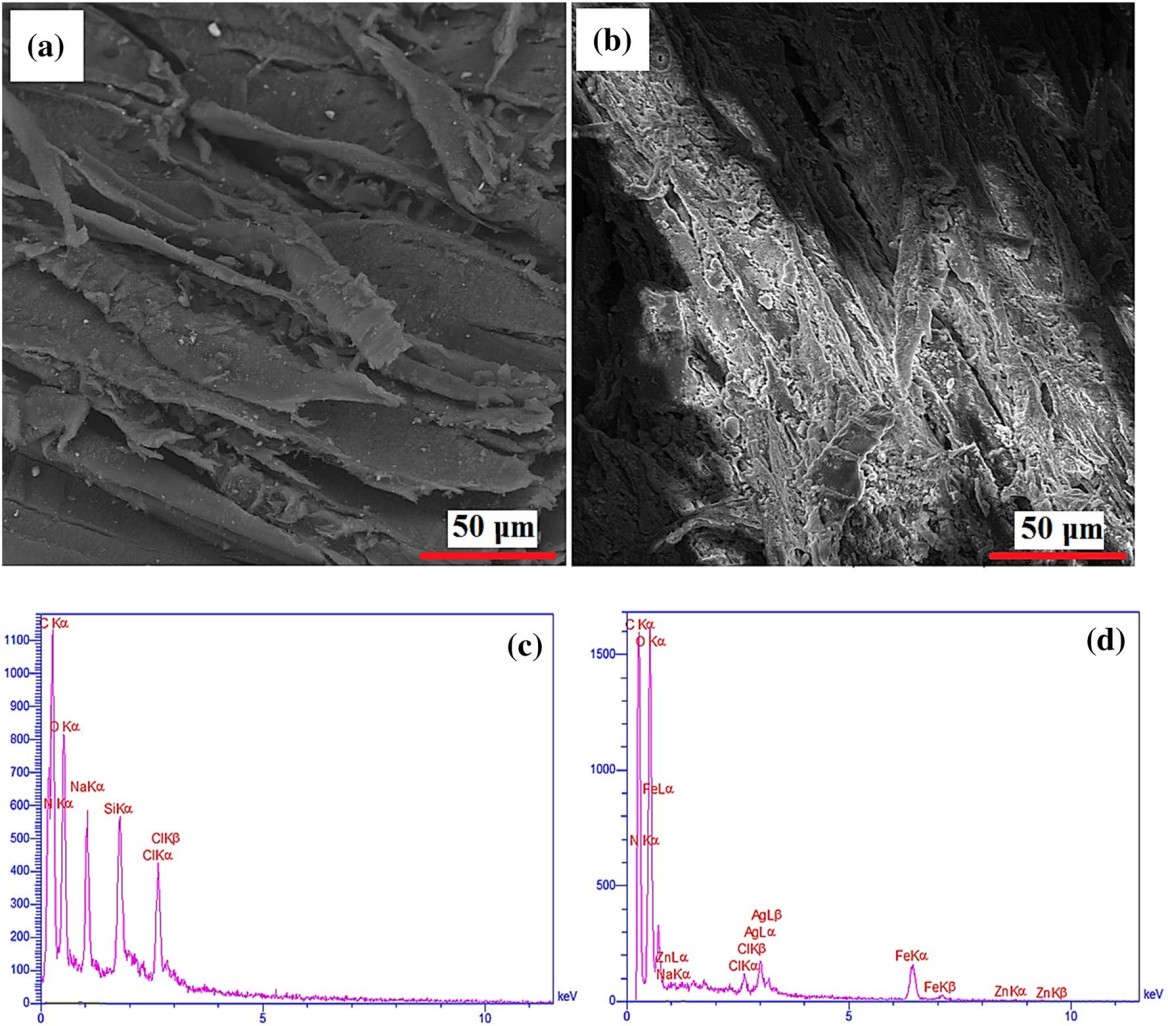
SEM and map analyses of raw lignocellulosic sawdust (**a**,**c**) and lignocellulosic sawdust-Fe/Zn composite (**b**,**d**).

**Figure 5:**
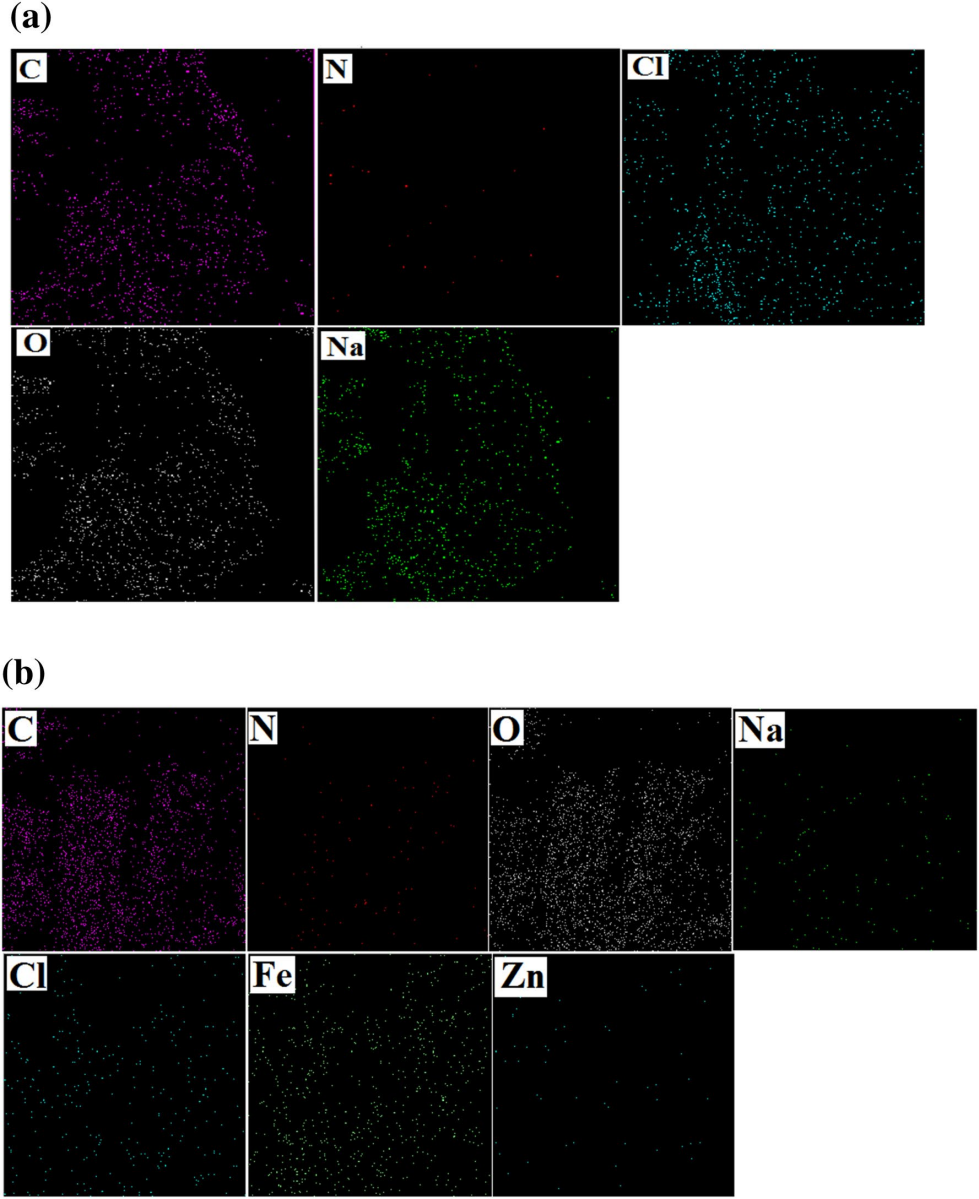
Maps of (**a**) raw lignocellulosic sawdust and (**b**) lignocellulosic sawdust-Fe/Zn composite.

A SEM image is one of the most widely used characterization techniques for identifying the morphology of prepared materials. Figure 4a shows the surface of the raw lignocellulosic sawdust. This verifies that raw lignocellulosic sawdust outer surface lacks porosity and is almost which might not be very effective in sorption systems^31^. After modification (Fig. 4b), it could be clearly seen that its surface became rougher, which might be due to the addition of FeCl_3_ and Zn(NO_3_) compared with raw lignocellulosic sawdust. Therefore, the surface of lignocellulosic sawdust-Fe/Zn became heterogeneous, which provided an excellent surface for dye adsorption. EDAX analysis was also conducted. In raw lignocellulosic sawdust (Fig. 4c), elements such as C, N, O, and Na were observed; however, after modification with FeCl_3_ and Zn(NO_3_)_2_ (Fig. 4d), new elements of Zn and Fe have appeared in the results. Carbon, hydrogen, oxygen, and nitrogen are the main chemical components of the lignin/cellulosic sawdust^32^. Similar findings were also reported by Khadir et al.^14^ and Li et al.^33^ once the surface of the raw material was coated by nanoparticles. In view of MAP results illustrated in Fig. 5, it can be observed that Zn and Fe elements were detected in the lignocellulosic sawdust-Fe/Zn composite. Hence, it is fair to suggest that the raw lignocellulosic sawdust was successfully supported by FeCl_3_ and Zn(NO_3_)_2,_ which might be beneficial in terms of dye adsorption/removal.

**Figure 6:**
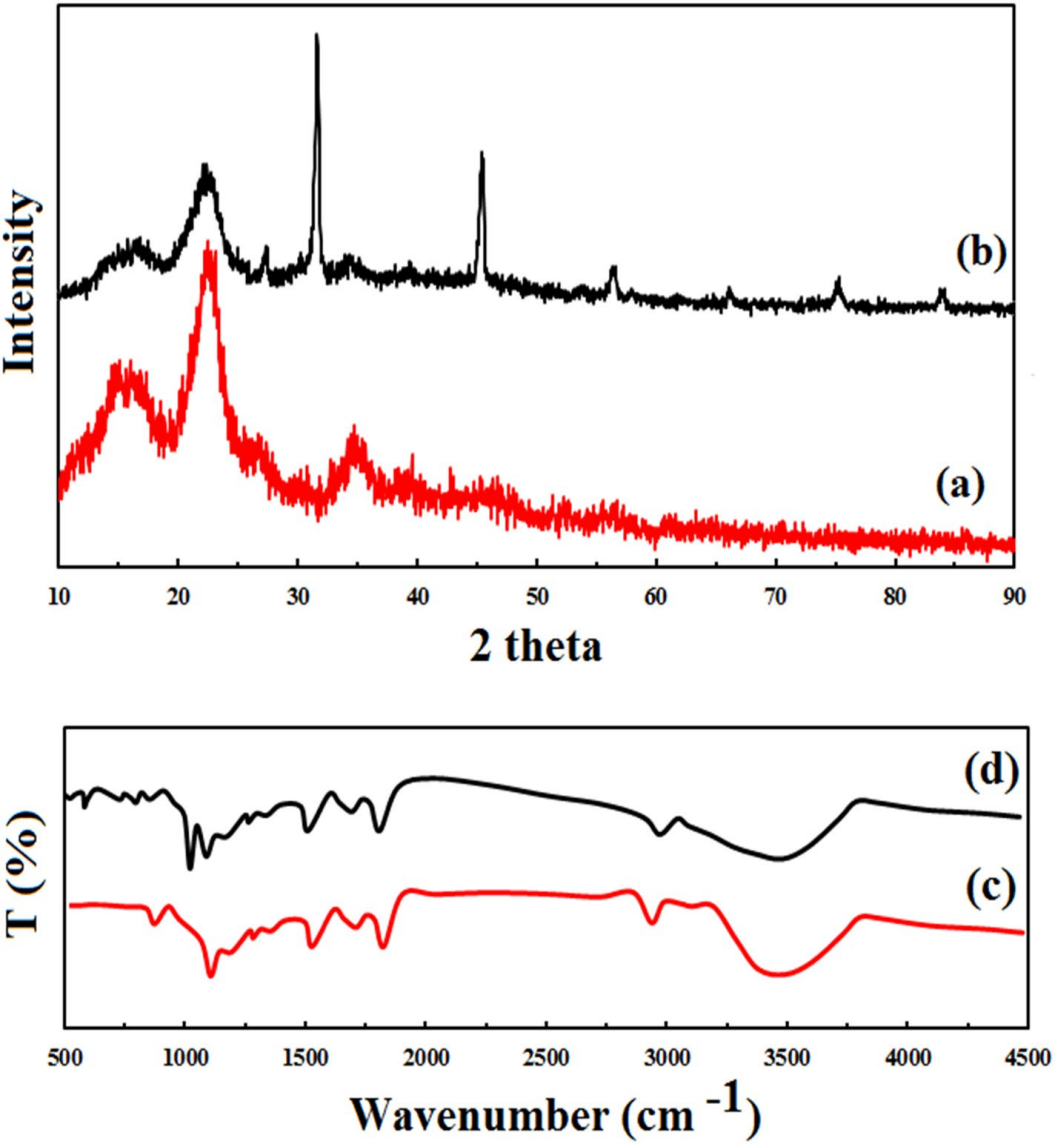
XRD and FTIR of raw lignocellulosic sawdust (**a**,**c**) and lignocellulosic sawdust-Fe/Zn composite (**b**,**d**).

The X-ray diffraction patterns of the raw lignocellulosic sawdust and lignocellulosic sawdust-Fe/Zn are illustrated in Fig. 6. As it is shown, there are two different peaks at 34°, 22° and 16°, which are indicative of the crystalline cellulose in the samples^23^. Regarding the nature of the material, observations of these peaks have already been expected. Chowdhury et al.^34^ also found identical peaks for the sawdust sample. After modification of raw lignocellulosic sawdust with Fe/Zn, new peaks appeared at 31°, 45°, 56°, 66°, 75°, and 84°. Similar peaks were seen in the other composites modified with Fe and Zn^30,35^. Figure 6 contains the FTIR spectra of raw sawdust and lignocellulosic sawdust-Fe/Zn nanocomposite. The broadband around 3476 cm^−1^ is attributed to the stretching of OH groups. The absorption band at 2920 cm^−1^ is due to C–H stretching^16^. The peak at 1511 cm^−1^ was assigned to the C=O bonds. C–OH bonds were observed at 1089 cm^−1^. In addition, C–H bending was detected at 856 cm^−1^^36^. After the modification, new peaks were emerged lower than 900 cm^−1^, including 585 and 730 cm^−1^ which are indicative of the presence of Fe/Zn nanoparticles^37^.

### Optimization procedure

#### The effect of contact time and comparison with raw lignocellulosic sawdust

**Figure 7:**
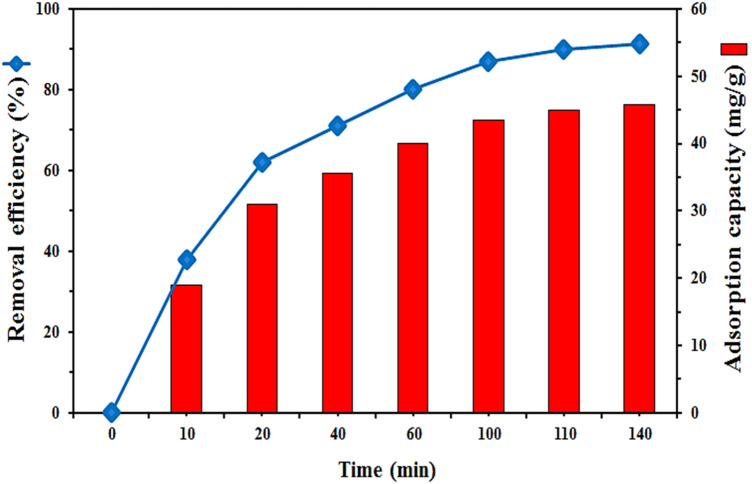
The effect of contact time on the removal efficiency of RO16 onto lignocellulosic sawdust-Fe/Zn bio-nanocomposite, and the adsorption capacity at different times (initial pH 4, 20 mg lignocellulosic sawdust-Fe/Zn bio-nanocomposite, 100 mL solution, initial RO16 concentration 10 mg L^−1^, and agitation speed 50 rpm).

Adsorption rate is one of the most important factors in batch adsorption experiments. To find an appropriate contact time that lignocellulosic sawdust-Fe/Zn bio-nanocomposite and RO16 reach equilibrium, 20 mg lignocellulosic sawdust-Fe/Zn bio-nanocomposite was added to the 100 mL solution of RO16 (10 mg L^−1^) at pH 4 and agitation speed of 50 rpm. Figure 7 shows the removal efficiency of the RO16 as a function of the contact time and the adsorption capacity of the adsorbent at different times. In both curves, RO16 elimination followed semi-identical trends.

As it is evident, during the first 20 min rapid dye uptake occurred due to the vacant adsorption sites available to remove the dye, reaching the removal efficiency and adsorption capacity of 62% and 31 mg g^−1^, respectively. As adsorption sites were filled gradually the dye removal efficiency increased at a slower pace. From 20 to 110 min, the adsorption capacity of the RO16 climbed from 31 to 45 mg g^−1^, and the removal efficiency also enhanced from 62 to 90.1%, respectively.

After 110 min, no obvious change in the removal efficiency, as well as the adsorption capacity was observed (the fluctuations were not significant). The equilibrium point, where adsorption and desorption rates are equal, signifies that the surface of the adsorbent is fully saturated and no further decolorization has occurred. Hence, the required time for reaching equilibrium was 110 min, and the following experiments were conducted with 110 min as the optimum contact time.

The synthesized lignocellulosic-based adsorbent in the current study exhibited the adsorption capacity of 45.2 mg g^−1^ toward RO16 molecules which was clearly superior to some of the previous studies investigating dye removal by biomaterials^38–40^. For instance, the application of lignin for decolorization was almost 17.5^41^, 8.5^42^, and 2.5^43^ times more effective than that of the coir pith, exhausted coffee ground, and surfactant modified laterite, respectively. This could be considered as one of the greatest advantages of the proposed material as well as its high removal efficiency.

**Figure 8:**
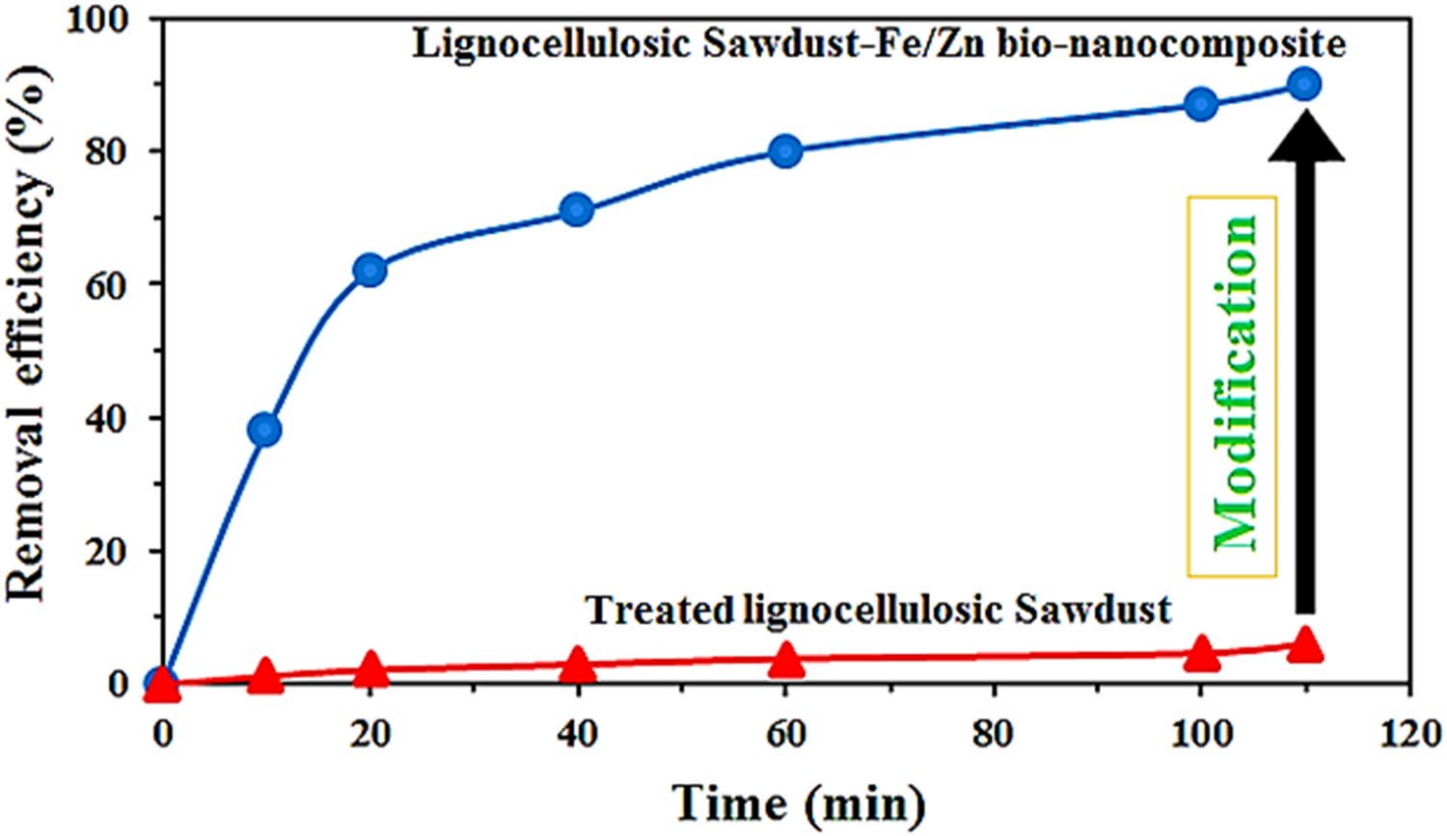
Comparison of Treated lignocellulosic sawdust and Lignocellulosic sawdust-Zn/Fe bio-nanocomposite for the removal of RO16 (initial pH 4, 20 mg Treated lignocellulosic Sawdust 100 mL solution, initial RO16 concentration 10 mg L^−1^, and agitation speed 50 rpm).

Figure 8 compares the efficiency of raw lignocellulosic sawdust and lignocellulosic sawdust-Fe/Zn bio-nanocomposite for the removal of RO16 under identical experimental conditions. The obtained results revealed that the performance of lignocellulosic sawdust-Fe/Zn bio-nanocomposite was 15.51 times higher than that of raw lignocellulosic sawdust in terms of RO16 adsorption. It proved that the modification conducted on lignin biomass resulted in promising results with excellent performance.

#### The effects of initial RO16 concentration and temperature

**Figure 9:**
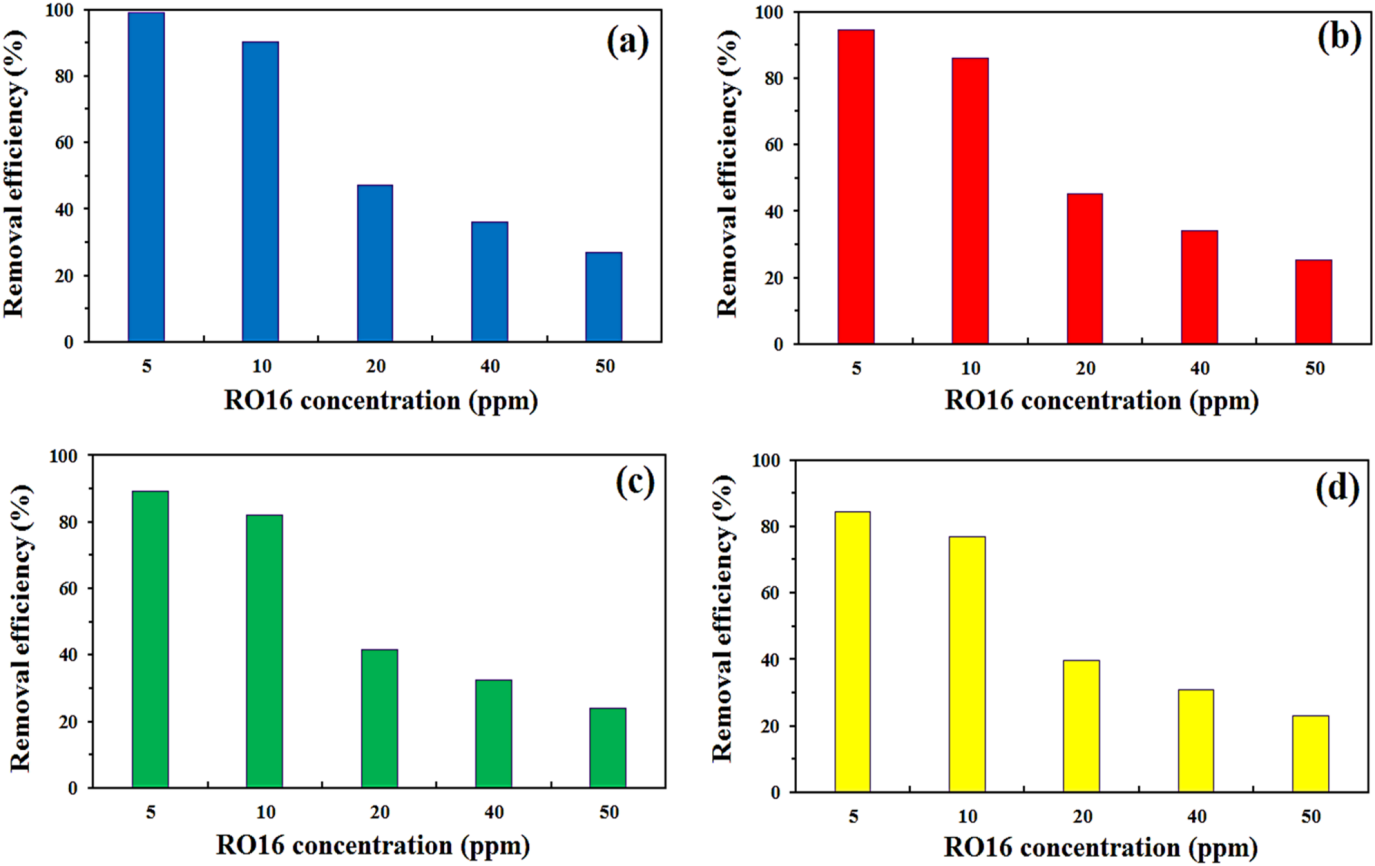
Effects of initial RO16 concentration and temperature (**a**) 25 °C, (**b**) 30 °C, (**c**) 35 °C and (**d**) 40 °C on the removal efficiency of the treatment process (initial pH 4, contact time 110 min, 20 mg lignocellulosic sawdust-Fe/Zn bio-nanocomposite, 100 mL solution, and agitation speed 50 rpm).

The concentration of pollutant molecules within the determined volume of aqueous solution adversely affects the efficiency of the sorption system. Pollutants could have a significant impact not only on adsorption processes but also on other treatment methods, such as advanced oxidation processes or biological systems.Accordingly, the effect of RO16 concentration ranging from 5 to 50 mg L^−1^ on the removal efficiency was investigated, and the results are illustrated in Fig. 9. At 25 °C, for instance, by increasing the pollutant concentration from 5 to 50 rpm, it was found that the RO16 removal efficiency decreased from 99.1 to 27.3%, respectively. In the case of other temperatures, a similar trend was observed. It looks that a high RO16 concentration could significantly deteriorate the adsorption efficiency. Previous studies also declared that for a wide range of pollutants, such as dye^44^, pharmaceuticals^45^ or heavy metals^46^, an increase in pollutant concentration resulted in a reduction in adsorption/removal efficiency. Such behavior could be ascribed to the fact that the number of vacant sites on lignocellulosic sawdust-Fe/Zn bio-nanocomposite is fixed during the experiments; however, the number of RO16 molecules are increasing. This leads to the absence of adequate sorption sites on the composite, culminating in lowering the efficiency. At this stage of optimization, RO16 concentration 5 mg L^−1^ was almost removed completely, however, to examine the effect of other parameters, the RO16 concentration was set at 10 mg L^−1^ for the following experiments.

Temperature influence was also paramount, and the results are depicted in Fig. 9. The effect of temperatures 25, 30, 35, and 40 °C was studied on RO16 removal efficiency. The temperature could have two different effects in different studies, in some it could be beneficial; however, for the other an increase in temperature could not be positive in terms of performance^47^. In the present study, it exhibited that at RO16 concentration 5 mg L^−1^, by fixing the temperatures at 25, 30, 35, and 40 °C, the removal efficiencies were 99.1, 94.3, 89.2, and 84.3%, respectively. Reducing the removal efficiency by increasing the temperature indicates that the process is exothermic in nature. In conclusion, 25 °C was chosen as the optimal value and the other experiments were conducted at this temperature. In view of industrial-scale treatment systems located in regions where the local temperature is around 25 °C, this result could be highly appreciated since there is no need to increase the temperature of the solution since at 25 °C, it has the best performance.

#### The effect of adsorbent dosage

**Figure 10:**
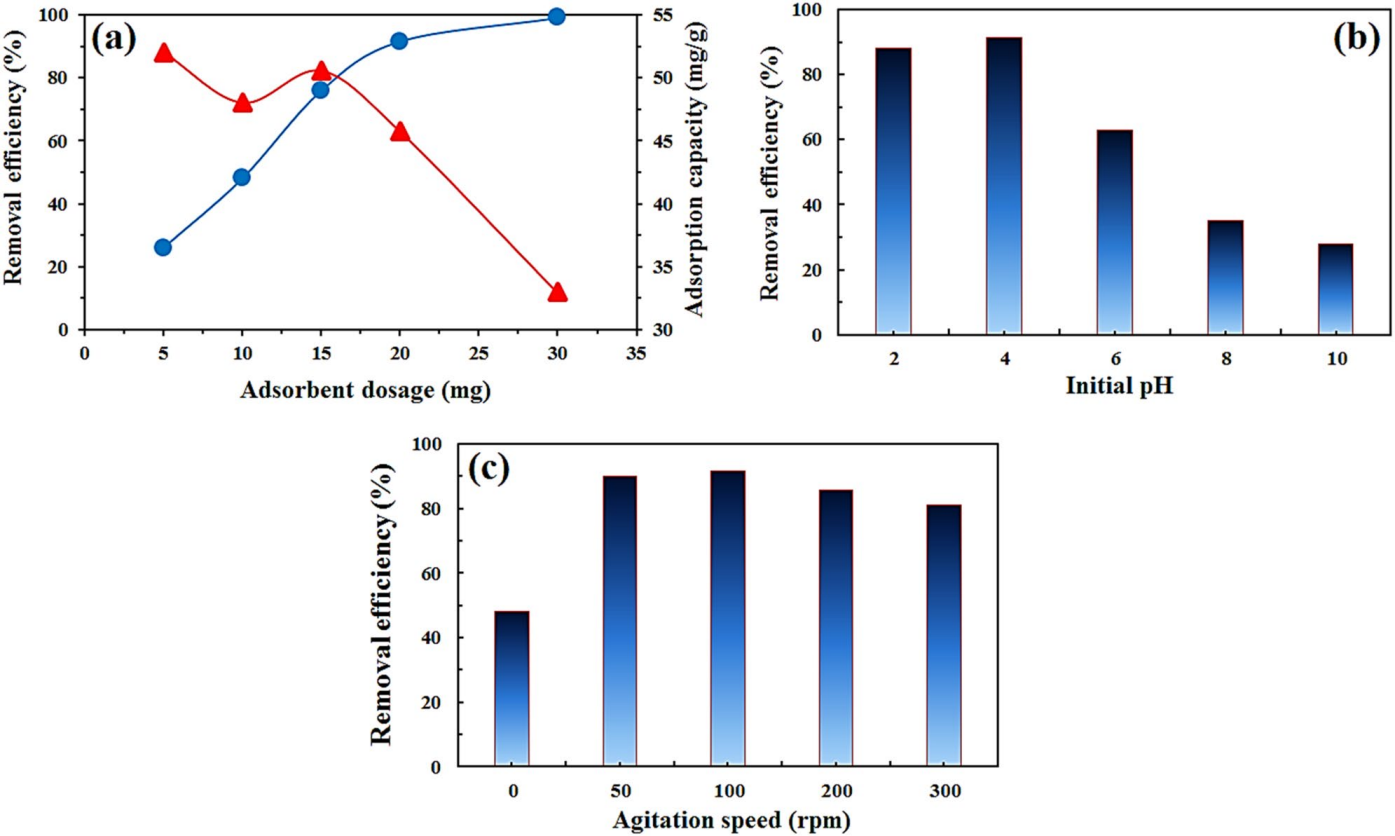
The effect of adsorbent mass (**a**), initial solution pH (**b**), and agitation speed (**c**) on RO16 removal by lignocellulosic sawdust-Fe/Zn bio-nanocomposite (contact time 110 min, RO16 concentration 10 mg L^−1^, and temperature 25 °C).

**Figure 11:**
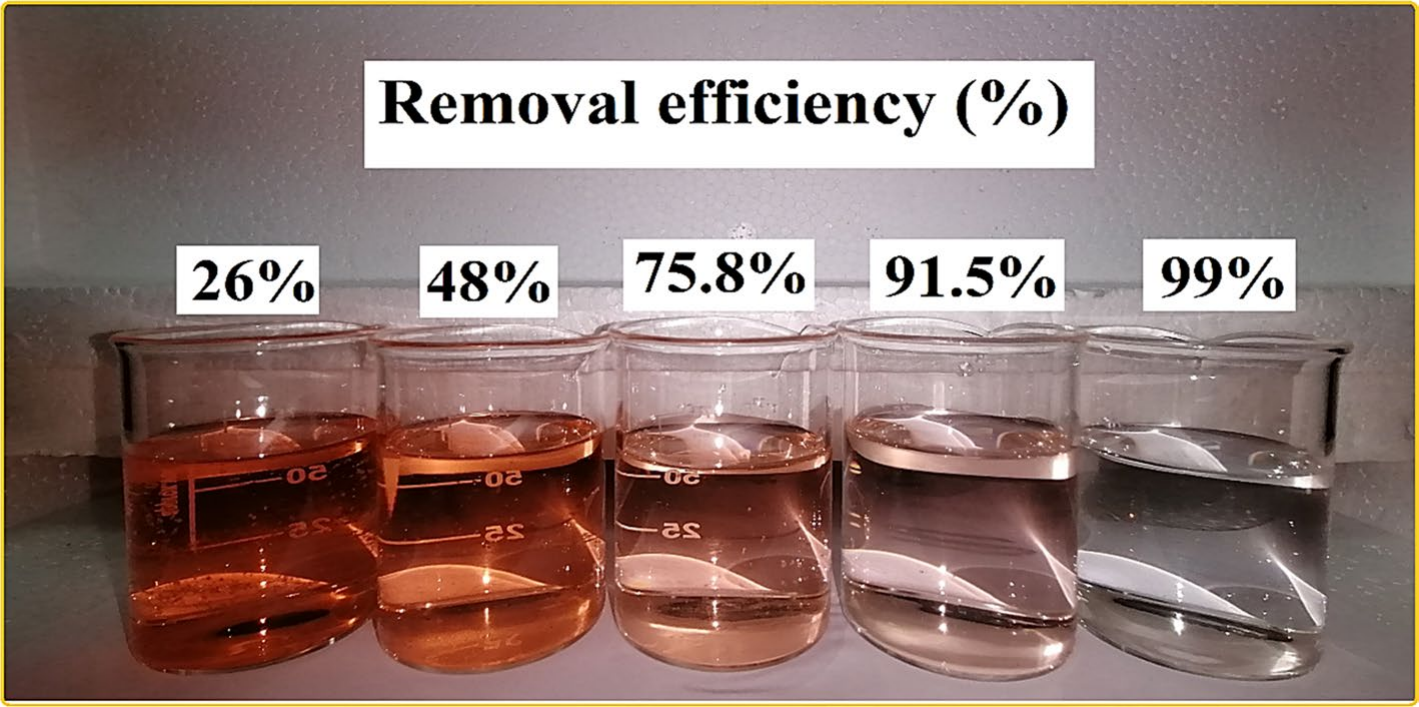
The color change of RO16 solution during purification by lignocellulosic sawdust-Fe/Zn bio-nanocomposite.

By controlling the adsorbent dosage, the active sites are available for adsorption. A low adsorbent dose results in few adsorption sites available to capture the sorbate; an increase in adsorbent concentration results in more binding sites and a higher dye removal rate^48^. The effect of adsorbent dosage at pH 4, time 110 min, and 10 mg L^−1^ reactive orange solutions was investigated. As Fig. 10a shows, the adsorbent dosage varies from 5 to 30 mg, increasing 5 mg each step. At the adsorbent dosage of 5 mg, the amount of lignocellulosic sawdust-Fe/Zn bio-nanocomposite in the solution was not sufficient to eliminate RO16 as a result the removal efficiency was only 26%. The removal efficiency of RO16 had sharply increased from 26 to 91.5%, by increasing the lignocellulosic sawdust-Fe/Zn bio-nanocomposite dosage from 5 to 20 mg, then from 20 to 30 mg the removal efficiency mildly climbed reaching 99%. Figure 11 shows the color change of RO16 solution during treatment, and it demonstrates that the water was almost completely decolorized. Therefore, 20 mg lignocellulosic sawdust-Fe/Zn bio-nanocomposite was used as an optimum adsorption dosage in the following experiments. The same trend was observed by Khadir et al.^31^ reported that the removal efficiency of RO16 skyrocketed from 47.2 to 97.3% with the adsorbent dosage from 50 to 100 mg. Other studies also have reported similar trends for the adsorbent dosage effect on orange dyes^49–51^. While the trend for the removal efficiency of RO16 was upward, the adsorption capacity conversely declined. When the adsorbent dosage varied from 5 to 10 mg, a decline followed by an increase in the adsorption capacity was noticed, from 15 to 30 mg lignocellulosic sawdust-Fe/Zn bio-nanocomposite, a steady decrease in the adsorption capacity from 50.53 to 33 mg g^−1^ occurred. The downward trend in the adsorption capacity when lignocellulosic sawdust-Fe/Zn bio-nanocomposite dosage increased from 5 to 30 mg was due to the competition among RO16 dye molecules for reaching the limited vacant sites^31^.

#### The effect of initial solution pH

pH is a prominent parameter in the adsorption process as it directly affects the ionization degree as well as the adsorbent surface charge. RO16 is an anionic dye that favorably adsorbs on positively charged surfaces^50^. Figure 10b illustrates the effect of pH on the removal efficiency of RO16. As the figure demonstrates, when the initial pH values increased from 2 to 10 (2 at each step), the removal efficiency decreased from 91.5 to 88.2, 63%, 35, to 28%, respectively. In the acidic environment, the surface of the Zn/Fe had positive charges so the electrostatic attraction between the positively charged lignocellulosic sawdust-Fe/Zn and negative RO16 molecules enhanced the adsorption of anionic dye. While at higher pH values the adsorbent surface neutralized and at the high alkaline environment the adsorbent surface charge became negative, so the negatively charged surface repulsed RO16 dye molecule. Based on the below reactions adsorption sites reached positive or negative electrical charges.

3 $$Zn-OH+{H}^{+} \leftrightarrow Zn-{{OH}_{2}}^{+}$$

4 $$Zn-OH+{H}^{-} \leftrightarrow Zn-{O}^{-}+{H}_{2} O$$

Similar results were obtained by some studies investigating the removal of anionic dyes^52–55^. Elimination of RO16, RO5, and Congo Red by zeolite-hydroxyapatite nanocomposite reached the maximum dye removal efficiency at pH 2^55^. Alver et al.^54^ reported that the reduction in the removal efficiency of dyes on zeolite was due to the ionic repulsion.

#### The effect of agitation speed

The agitation speed plays a major part in controlling the adsorption process; usually faster agitation speeds enhance adsorption as they facilitate ion mobility in solution, which is the key to effective collisions between adsorbents and adsorbates. When mass transfer across the boundary layer is one of the rate-controlling processes, stirring affects adsorption. As Fig. 10c demonstrates the trend, during the early stages of the adsorption process higher stirring speed resulted in higher removal efficiency. As agitation speed increased from 0 to 100 rpm with an interval of 50 rpm the removal efficiency of RO16 dye molecules rose from 48 to 91.5%. The further increase of agitation speed from 100 to 200 to 300 rpm was associated with lower dye uptake, this reduction can be attributed to the departure of RO16 molecules from the lignocellulosic sawdust-Fe/Zn bio-nanocomposite adsorption sites because high stirring speed could overcome the electrostatic attraction between the adsorbent and RO16 molecules. A similar trend was observed for Methylene Blue uptake onto Apricot stone activated carbon with various agitation speeds from 100 to 1200 rpm. The maximum removal efficiency of MB was achieved at a speed of 300 rpm. At higher stirring speed, the mg of MB adsorbed on the adsorbent decreased mildly as higher speed made it harder for MB to be electrostatically attracted to the sorbent^56^. Munagapati et al.^52^ studied the effect of agitation speed (50–400 rpm) on the adsorption of Reactive Black 5 by modified banana peel powder. They found the adsorption capacity of RB5 increased from the agitation speed of 50 to 200 rpm, and from 200 to 400 rpm it stayed constant due to the disruption of immobilized particles. Hence, 100 rpm was chosen as the optimum one.

### Kinetics of adsorption process

To design an adsorption system, the rate at that adsorption is underlying because the kinetics of adsorption disclose the adsorption mechanism. Some reaction kinetics and diffusion models are employed to get an insight into the adsorption mechanism^57^. The experimental data were fitted using the pseudo-first-order (PFO) and pseudo-second-order (PSO), these models were evaluated to identify the adsorption process of RO16 dye onto lignocellulosic sawdust-Fe/Zn bio-nanocomposite. The linear forms of the two studied reaction kinetic models are expressed as Eqs. (5) and (6):

5 $$\mathit{ln}\left({q}_{e}-{q}_{t}\right)=-{K}_{1}t+ln{q}_{e}$$

6 $$\frac{t}{{q}_{t}}=\frac{1}{{K}_{2}{q}_{e}^{2}}+\frac{1}{{q}_{e}}t$$

**Figure 12:**
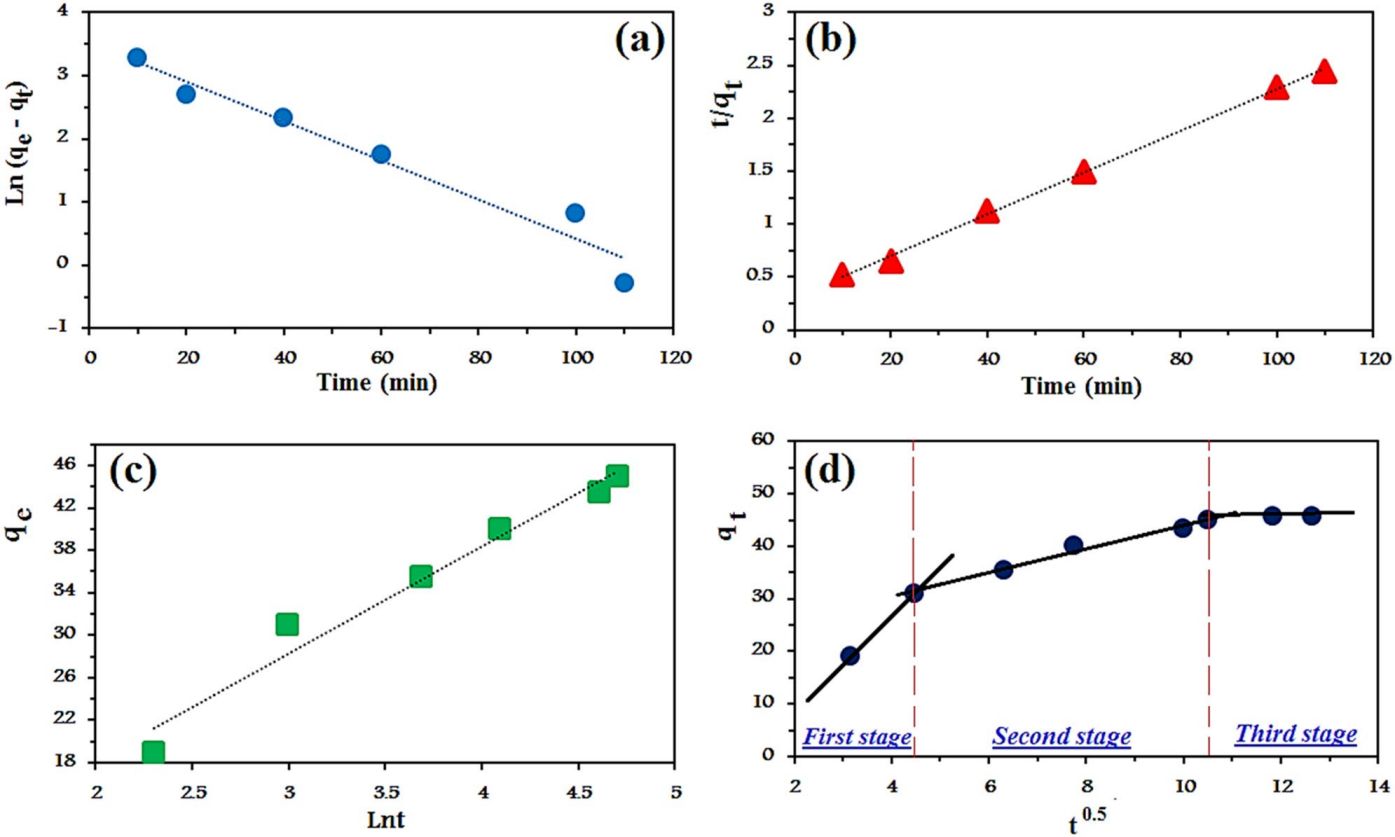
Linear kinetic models of (**a**) PFO (**b**) PSO (**c**) Elovich, and (**d**) intra-particle diffusion for RO16 adsorption by lignocellulosic sawdust-Fe/Zn bio-nanocomposite.

The linear plots of PFO and PSO are shown in Fig. 12 and the calculated parameters are listed in Table 1. In order to determine the best appropriate model, the coefficient of determination (R^2^) and the adsorption capacities were compared with each other. Although for both models, R^2^ values were high, PSO possessed a higher value and much more nearer to unity. In addition, the calculated adsorption capacity based on PSO was in good alignment with experimental adsorption capacity than the results of PFO. In conclusion, it is fair to suggest that PSO is the most excellent equation describing the adsorption of RO16 by lignocellulosic sawdust-Fe/Zn bio-nanocomposite. This confirmed that chemisorption is dominant for the removal of RO16 dye. Functional groups on the lignocellulosic sawdust-Fe/Zn bio-nanocomposite are likely to establish chemical bonds with RO16 molecules.

**Table 1:** Kinetic constants of RO16 adsorption by lignocellulosic sawdust-Fe/Zn composite.

Models	Parameters	RO16
PFO	${K}_{1 }$(${\mathrm{min}}^{-1}$)	0.031
	${q}_{e}$ (mg g^−1^)	33.91
	${R}^{2}$	0.9570
PSO	${K}_{2}$ (g ${\mathrm{mg}}^{-1} {\mathrm{min}}^{-1}$)	0.00126
	${q}_{e}$ (mg g^−1^)	50.76
	${R}^{2}$	0.9982
Elovich	*β* (g mg^−1^)	0.098
	*α* (mg g min^−1^)	10.02
	${R}^{2}$	0.9693
Intra-particle	${K}_{i}$ (mol kg^−1^ ${\mathrm{min}}^{0.5}$)	C (mol kg^−1^)
First stage	9.16	−9.97
Second stage	2.18	21.19
Third stage	0.368	41.21

Many former investigations have also proved the suitability of PSO assumptions for dye removal. Khadir et al.^31^ stated that Reactive Orange 5 removal reached the equilibrium state at 120 min and the kinetics data followed PSO due to high R^2^ value. In another study conducted by Cherifi et al.^58^ for dye removal, PSO was introduced as the accurate model, explaining the time results.Gupta et al.^59^, and Xu et al.^60^ also demonstrated that the kinetic data were fitted to the pseudo-second-order kinetics. Motamedi et al.^61^ deduced that PSO assumptions are in many cases superior to PFO. The inapplicability of PFO model could be justified by its limitations, including difficulty in selecting q_e_ value or inability to describe the whole process.

The Elovich or Roginsky–Zeldovich equation is another commonly used model in adsorption studies which is expressed as Eq. (7):

7 $${q}_{e}= \frac{1}{\upbeta } Ln t+\frac{1}{\upbeta } Ln (\alpha \beta )$$

where $\alpha$ (g mg^−1^) and β (mg g^−1^ min^−1^) are the Elovich constants. These parameters are found from the linear plot of q_e_ versus ln t.

Considering the obtained results, R^2^ (0.9693) value of the Elovich model was higher than PFO and lower than PSO model. Although PSO is undoubtedly the best model, Elovich assumptions exhibited that the lignocellulosic sawdust-Fe/Zn bio-nanocomposite is heterogeneous and RO16 molecules build up on the sorbent with means of chemical bonds.

As Wang and Wang^62^ stated, adsorption process is involved multistep phenomena, including the movement of the adsorbate molecules from the bulk solution to a region surrounding the adsorbent, external surface, and finally the interior pores. To determine the rate-limiting step, the intraparticle diffusion model proposed by Weber and Morris could be employed written as Eq. (8)^63^:

8 $${q}_{t}={K}_{i}{t}^{0.5}+C$$

where K_i_ is the intraparticle diffusion rate constant (mg g min^0.5^) and C is a constant for any experiment (mg g^−1^). The plot of the model is illustrated in Fig. 12d.

As a result, the plot consists of three lines without passing through the origin. Several mechanisms are involved in the RO16 dye removal by lignocellulosic sawdust-Fe/Zn bio-nanocomposite. Essentially, RO16 molecules must first pass the boundary layers around the lignocellulosic sawdust-Fe/Zn bio-nanocomposite, then move toward the external surfaces, and finally enter the pores. In the equilibrium phase, the adsorption and desorption rates equal each other, as indicated by the plateau line. Hence, it is concluded that the sorption process is controlled by film diffusion and intraparticle diffusion. The plot unveiled that a rapid RO16 dye adsorption onto lignocellulosic sawdust-Fe/Zn bio-nanocomposite happened in the first 20 min, and then it slowed down in the following mind. Boundary layer diffusion or macro-pore diffusion were reasons of such rapid sorption, and the slow adsorption was due to the intraparticle diffusion or micro-sore diffusion. Pholosi et al.^64^, Khadir et al.^14^ and Pan et al.^65^ reported similar observations.

Furthermore, intraparticle diffusion model could determine the initial adsorption behavior of the system by Eq. (9):

9 $${q}_{ref}={K}_{i}{t}_{ref}^{0.5}+C$$

where q_ref_ and t_ref_ are the maximum adsorption capacity (mg g^−1^) and the maximum adsorption time (min), respectively. By subtracting former equation by intraparticle model and then rearrangement yields:

10 $${q}_{ref}-{q}_{t}={K}_{i}({t}_{ref}^{0.5}-{t}_{0.5})$$

11 $$\left(\frac{{q}_{t}}{{q}_{ref}}\right)=1-{R}_{i}\left[1-{\left(\frac{t}{{t}_{ref}}\right)}^{0.5}\right]$$

where R_i_ is defined as Eq. (12):

12 $${R}_{i}=\frac{{q}_{ref}-C}{{q}_{ref}}=1-\frac{C}{{q}_{ref}}$$

R_i_ is called the initial factor based on intraparticle diffusion, and based on its values, it has five possibilities:

1. R_i_ = 1 → No initial adsorption
2. 0.9 < R_I_ < 1 → Weakly initial adsorption
3. 0.5 < R_i_ < 0.9 → Intermediately initial adsorption
4. 0.1 < R_i_ < 0.5 → Strongly initial adsorption
5. R_i_ < 0.1 → Approaching completely initial adsorption

**Figure 13:**
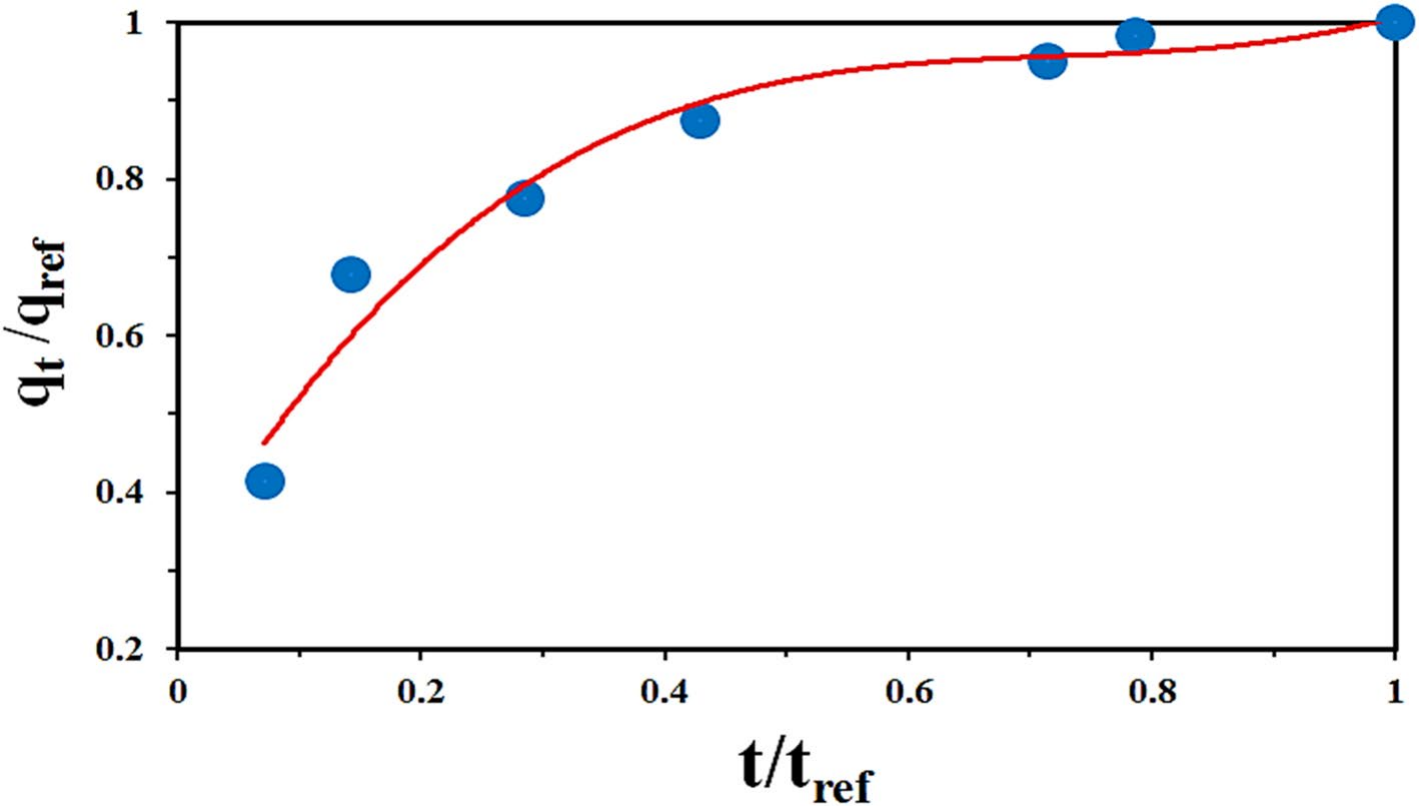
Dimensionless characteristic curves of the adsorption of RO16 by lignocellulosic sawdust-Fe/Zn bio-nanocomposite.

In the case of current investigation, Fig. 13 shows the dimensionless characteristic curve of the adsorption of RO16 by lignocellulosic sawdust-Fe/Zn bio-nanocomposite.

### Isotherms of adsorption

**Figure 14:**
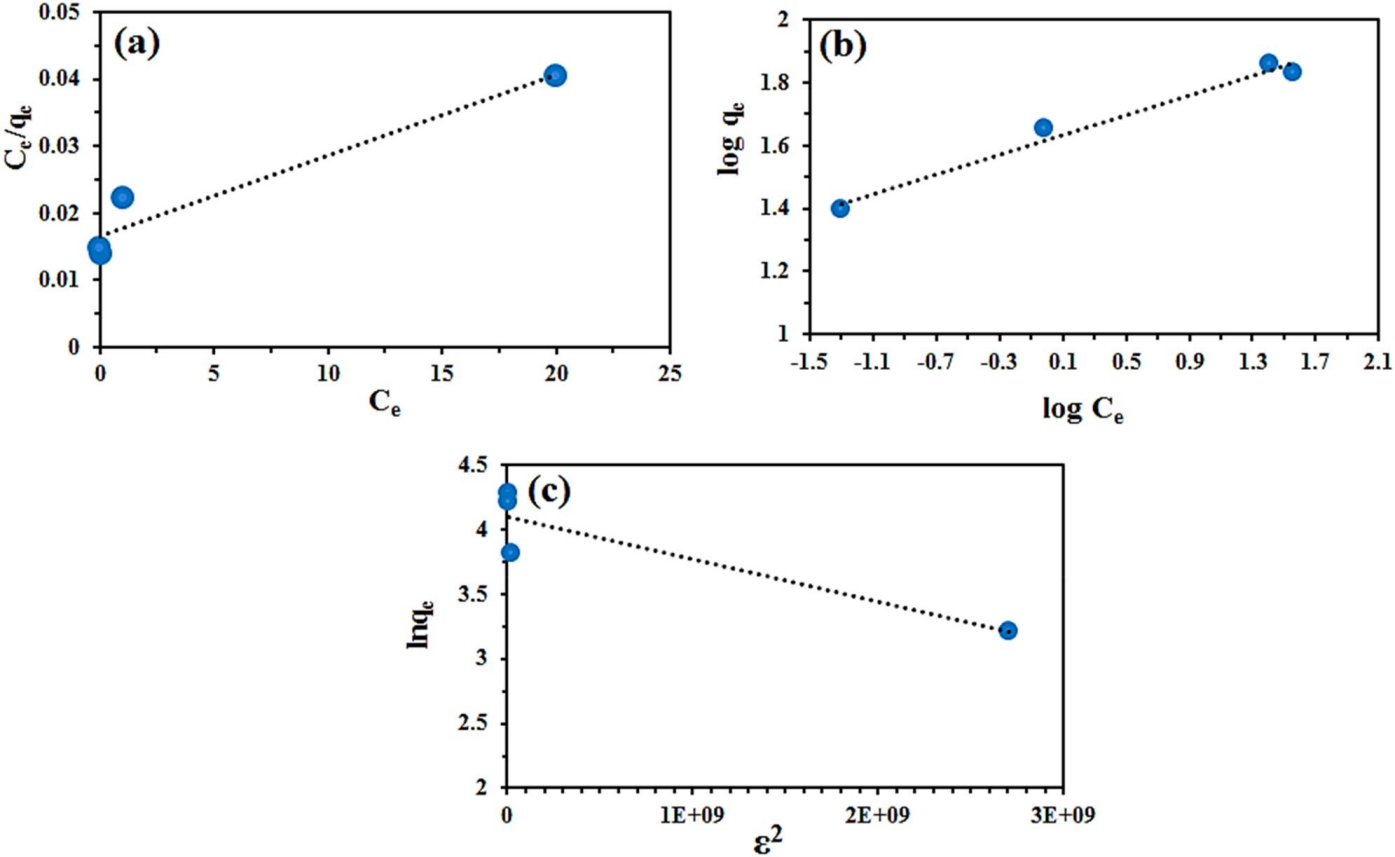
The linear plots of Langmuir (**a**), Freundlich (**b**), and DR (**c**) for the removal of RO16 by lignocellulosic sawdust-Fe/Zn.

It is possible to determine the surface properties and affinity of an adsorbent by analyzing the isotherms` parameters. Adsorption equilibrium equations relate dye adsorbed on lignocellulosic sawdust-Fe/Zn bio-nanocomposite to dye concentration in solution at equilibrium. A Langmuir and Freundlich model was fitted to the experimental data. A Langmuir model assumes monolayer adsorption on a homogeneous surface, while a Freundlich isotherm describes multilayer adsorption on a heterogeneous surface^48^. Dubinin–Radushkevich (DR) has also gained attention of scholars as semiempirical temperature-dependent model which is based on the pore-filling^66^. The linear forms of Langmuir, Freundlich, and DR models can then be defined by Eqs. (13–15), respectively^67^:

13 $$\frac{{C}_{e}}{{q}_{e}}=\frac{{C}_{e}}{{q}_{m}}+\frac{1}{{q}_{m}{K}_{L}}$$

14 $$\mathrm{log}{q}_{e}=\mathrm{log}{K}_{F}+\frac{1}{n}\mathrm{ log}{C}_{e}$$

15 $$ln{q}_{e}=ln{q}_{m}-\beta {\varepsilon }^{2}$$

where C_e_ (mg L^−1^) is the equilibrium concentration of RO16, q_e_ (mg g^−1^) is the equilibrium adsorption capacity, q_m_ (mg g^−1^) is the maximum adsorption capacity of dye on lignocellulosic sawdust-Fe/Zn, and K_L_ (L g^−1^) is Langmuir constant. K_F_ (mg g^−1^) and n are Freundlich adsorption isotherm constant, and heterogeneity of adsorption process, respectively.$\beta$ (mol^2^ kJ^−2^) depicts a constant related to the adsorption energy and $\upvarepsilon$ (kJ mol^−1^) is the desorption potential. The linear plots and calculated constants are illustrated and tabulated in Fig. 14 and Table 2, respectively.

**Table 2:** Isotherm constants of RO16 adsorption by lignocellulosic sawdust-Fe/Zn composite.

Models	Parameters	RO16
Langmuir	${q}_{m}$ (mg g^−1^)	60.97
	${k}_{l}$ (L mg^−1^)	13.66
	${R}^{2}$	0.9343
Freundlich	${k}_{f}$ $((\text{mg}\,\text{g}^{-1}){(\frac{\mathrm{L}}{\mathrm{g}})}^{\frac{1}{\mathrm{n}}})$	40.73
	${n}_{f}$	6.38
	${R}^{2}$	0.9758
D–R	*ẞ* (${\mathrm{mol}}^{2}$ kJ^−2^)	3 × ${10}^{-10}$
	${q}_{m}$ (mg g^−1^)	60.34
	${R}^{2}$	0.8271

Coefficient of determination (R^2^) was considered in determining the best model. Among the investigated isotherm models, Freundlich exhibited higher R^2^ value compared to Langmuir or DR. n value of Freundlich was found 6.38, indicating that the isotherm is satisfactory, since it is greater than one^55^. In conclusion, it can be expresses that multilayer of RO16 dye molecules tend to be formed on the heterogeneous surface of the lignocellulosic sawdust-Fe/Zn composite. Many scholars believed that Freundlich assumptions generally fit the chemical adsorption (note that n value is of great importance)^68–70^. By chemisorption, lignocellulosic sawdust-Fe/Zn makes chemical bonds with dye molecules via a significant number of functional groups. As a matter of fact, chemisorption could be considered the dominant sorption mechanism.

### Thermodynamic parameters

Thermodynamic parameters are generally employed to determine the feasibility, spontaneity, and nature of the sorption process by means of Gibbs free energy ΔG° (J mol^−1^) enthalpy ΔH° (J mol^−1^) and entropy ΔS° (J mol^−1^ K^−1^). The following relation could be stated with the thermodynamic equilibrium constant and other thermodynamic parameters (Eqs. 16, 17):

16 $$\mathrm{ln}\left({K}_{ad}\right)=\mathrm{ln}\left(1000\times \frac{{q}_{e}}{{C}_{e}}\right)=\frac{\Delta S^\circ }{R}-\frac{\Delta H^\circ }{RT}$$

17 $$\Delta G^\circ =\Delta H^\circ -T\Delta S^\circ $$

The results are tabulated in Table 3. Accordingly, the negative value of ΔH° means that the whole process is exothermic, and lignocellulosic sawdust-Fe/Zn performed better at lower temperature. The experimental findings also demonstrated that 25 °C was the best temperature among the others in terms of dye removal. The negative values of ΔG° at different temperatures indicate that the adsorption of RO16 on lignocellulosic sawdust-Fe/Zn is feasible and the adsorption process is spontaneous. Negative value of ΔS° suggested that RO16 removal was enthalpy driven.

**Table 3:** Thermodynamic parameters of RO16 adsorption by lignocellulosic sawdust-Fe/Zn composite.

T (°C)	− ΔG° (J mol^−1^)	− ΔH° (kJ mol^−1^)	−ΔS° (J mol^−1^ K^−1^)
25	26,871.9	57.74	103.60
30	26,353.9		
35	25,835.9		
40	25,317.9		

### Comparison with other adsorbents

Several adsorbents have been used for dyes removal until now. Herein, in Table 4 the removal rate of RO16 via lignocellulosic sawdust-Fe/Zn composite was compared with previous reports. As it is shown, the percentage of removal for sawdust-Fe/Zn composite displayed higher rate of elimination compared with other cited adsorbents. Thus, the developed adsorbent could be considered as a prospective candidate for the efficient removal of RO16 from their aqueous solutions higher than that of the majority of the adsorbents reported in the literature.

**Table 4:** Comparison with other adsorbents for the removal of RO16.

Adsorbent	Removal efficiency (%)	Adsorption capacity (mg g^−1^)	References
MRL–Co/Al LDH	97	53.04	^71^
C_3_N_4_/MgO/Vis	82	–	^72^
Activated carbon prepared from rice husk ash	–	13.32	^73^
AC@nZVI/Ni	–	10.53	^74^
m-Cs-PVA/FA	90.3	123.8	^75^
Arachis hypogaea pod powder	–	56.48	^76^
MgO/g-C_3_N_4_/zeolite nanocomposite	94.09	–	^77^
ZIF-8@Fe_3_O_4_@BNT	98.5	40.5	^78^
Thiourea-Fe_3_O_4_-TiO_2_ modified chitosan	82	133.69	^79^
Activated carbon derived from the stems of Phyllanthus reticulatus plant	85.1	–	^80^
Lignocellulosic sawdust-Fe/Zn	99	60.97	This work

## Conclusion

According to the present study, a low-cost non-toxic bio-nanocomposite, lignocellulosic sawdust-Fe/Zn, could effectively remove RO16 dye from an aqueous solution. SEM/EDAX, FTIR, and XRD results showed that the composite was successfully prepared. By increasing the contact time, the removal efficiency increased until equilibrium was reached. Additionally, decreasing dye concentration and increasing adsorbent dosage can both improve sorption efficiency and performance. At lower temperatures, the most efficient removal occurred, and thermodynamic results indicated that RO16 dye adsorption could occur spontaneously. When the conditions were optimal, nearly all dye molecules were removed from the aqueous solution. Kinetics and isotherms revealed that PSO and Freundlich were excellent models, showing that RO16 molecules tend to stabilize chemical bonds with functional groups of lignocellulosic sawdust-Fe/Zn composite. Surprisingly, lignocellulosic sawdust-Fe/Zn bio-nanocomposite significantly performed better than raw sawdust. In short, lignocellulosic sawdust-Fe/Zn composite is a promising, cost-effective, and non-toxic material with significant performance in terms of dye removal.

## Data Availability

All data generated or analyzed data for experimental part during this study are included in this published article. The data that support the findings of this study are available from the corresponding author, [Mehrdad Negarestani], upon reasonable request. Moreover, all other data that support the plots within this paper and other findings of this study are available from the corresponding author upon reasonable request.
